# Using simulated patient methodology to assess sick day guidance in community pharmacy: The case of an elderly patient with diabetes

**DOI:** 10.1016/j.rcsop.2025.100623

**Published:** 2025-06-11

**Authors:** Tristan Coppes, Hetty Prins, Kees A. van Amerongen, Teun van Gelder, Ellen S. Koster, Marcel L. Bouvy

**Affiliations:** a Department of Pharmacoepidemiology and Clinical Pharmacology, Utrecht Institute for Pharmaceutical Sciences (UIPS), Faculty of Science, Utrecht University, Utrecht, the Netherlands; b Onderzoeksprogramma Kwaliteit Apotheken (Apotheken Monitoring Program), Vught, the Netherlands; c Department of Clinical Pharmacy & Toxicology, Leiden University Medical Centre, Leiden, the Netherlands; d Education Center, University Medical Center Utrecht, Utrecht University, Utrecht, the Netherlands

**Keywords:** Sick day guidance, Community pharmacy, Self-care, Dehydration, Diarrhoea, Simulated patient

## Abstract

**Background:**

Certain high-risk medication, such as diuretics, should be temporarily adjusted during sick days (diarrhoea, vomiting or fever) to reduce the risk of adverse drug events. Guidelines refer to this as ‘sick day guidance’. Prior research has shown limited awareness among pharmacy staff of these recommendations.

**Objective:**

This study assessed how community pharmacies in the Netherlands address a simulated self-care inquiry related to diarrhoea from a 71-year-old patient with diabetes.

**Methods:**

Three trained simulated informal caregivers visited community pharmacies unannounced to seek an antidiarrheal product for a 71-year-old family member with diabetes and high-risk medication. Data were collected through a data collection form with audio recording.

**Results:**

A total of 64 pharmacies were visited. Current comorbidities and medications were identified in 59 % (38/64) of the pharmacies. Sixteen out of 64 pharmacies (25 %) provided sick day guidance either through temporary medication adjustment or GP referral. In more than 80 % of the pharmacies, a pharmaceutical product was dispensed, most often loperamide. The pharmacies that did not identify current comorbidities and medications (*n* = 26), did not provide sick day guidance nor referred to the GP.

**Conclusions:**

Sick day guidance was applied in one in four cases. Identifying current comorbidities and medications is essential for providing sick day guidance.

## Highlights

• Identification of who was experiencing diarrhoea was performed in 70 % of the cases.
• The risk of dehydration due to diarrhoea was mentioned in 50 % of the cases.
• One in four pharmacies provided sick day guidance for a diarrhoea self-care inquiry.
• Sick day guidance was only provided if comorbidities and medications were identified.

## 1 Introduction

Community pharmacies play a key role in self-care for minor ailments by providing medical advice and, if needed, dispensing self-care products.^1^ Conducting a patient consultation is crucial to identify comorbidities and medications, especially in elderly patients at risk of dehydration, for example, due to diarrhoea, vomiting or high fever (so-called ‘sick days’) or heatwaves. For these patients, continued concomitant use of Sulfonylureas, ACE inhibitors, Diuretics, Metformin, Angiotensin receptor blockers, NSAIDs and SGLT2 inhibitors (SADMANS)during sick days can cause adverse drug events or acute kidney injury.^2^^,^^3^ Clinical guidelines increasingly recommend “sick day guidance”, thus advising temporary adjustment or discontinuation of SADMANS drugs to mitigate these risks.^4^

In the Netherlands, the Royal Dutch Pharmacy Association (KNMP) developed self-care guidelines to assist community pharmacists and pharmacy technicians in providing adequate self-care advice.^5^ For diarrhoea in patients older than 70 years, or patients having impaired renal function, cardiovascular disease or diabetes, the guidelines recommend always referring to a general practitioner (GP) and considering and discussing temporary adjustment of any of the SADMANS medications.

However, research has shown that sick day guidance implementation is not widespread among patients and healthcare professionals.^4^^,^^6^ Additionally, barriers exist in providing recommendations for adequate self-care in community pharmacies such as high work pressure and limited knowledge about sick day guidance among healthcare professionals.^7^ It is unclear whether pharmacies in the Netherlands apply sick day guidance or refer to a GP in the case of sick days in high-risk patients. Therefore, this simulated patient study assessed how pharmacies in the Netherlands manage a self-care inquiry related to diarrhoea in a patient at high risk for adverse drug events.

## 2 Methods

### 2.1 Setting

In May and June 2023, the Apotheken Monitoring Program (AMP) research team conducted a simulated patient study. Pharmacies in the Netherlands can subscribe to AMP's simulated patient service. The pharmacies pay a yearly fee and four times a year, a simulated patient study is conducted by AMP on a certain topic. The topics are self-care or prescription-related. A trained simulation patient visits the pharmacy and scores the pharmacy on several domains such as contact with the patient, privacy, WWHAM questions (Who is it for, What are the symptoms, How long have the symptoms been present, has any Action been taken, what Medications are in use), information provision, advice, dispensing and conclusion. An extensive report summarises the experience at the pharmacy and compares it with the average and best-performing pharmacy and, if applicable, with previous results.

### 2.2 Design

**Fig. 1:**
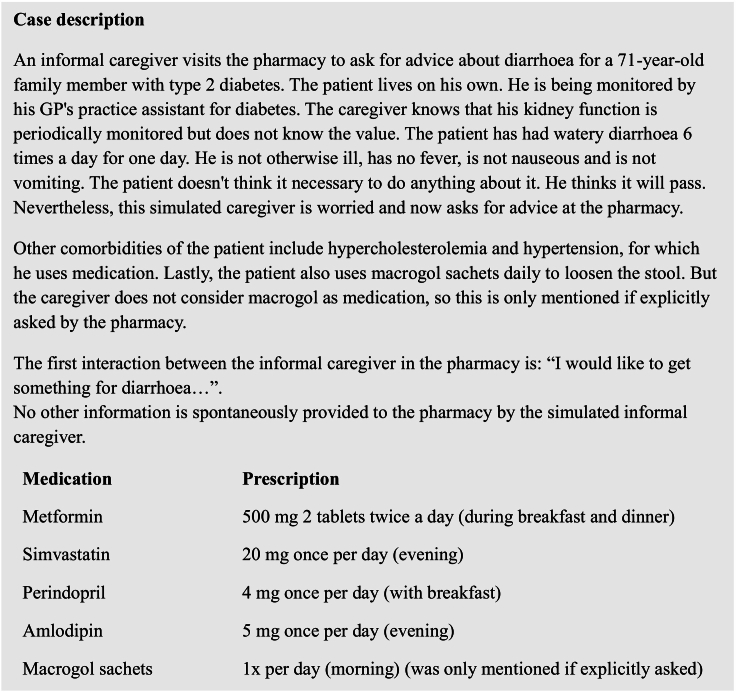
Case description used by the trained actor in this study.

In this simulated patient study, a simulated informal caregiver visited community pharmacies for a self-care question related to diarrhoea of an elderly family member. Beforehand, a detailed case description, see Fig. 1, was developed and clinical guidelines were consulted to define the appropriate recommendation. Additionally, the case included background information about the elderly relative such as comorbidities, current complaints and answers to anticipated questions by the pharmacy (see Appendix A). The case was pilot-tested in a pharmacy and improved. The topic of the visit is announced before the visit to the pharmacists. This included general information on the topic (diarrhoea) and reference to the current relevant clinical guidelines regarding diarrhoea. The pharmacist was not informed that sick day guidance required attention during the mystery guest visit. The pharmacist can decide whether to reveal the topic to the team beforehand. However, the exact timing of the visitation of the simulated informal caregiver was not announced.

#### 2.2.1 Data collection

A total of three trained and certified simulation informal caregivers individually visited the pharmacies. To minimise variability between the simulated informal caregivers, an extensive conversation protocol was developed, and an online 2-h video-training session was held. Every pharmacy was visited once face-to-face by the simulated informal caregiver. Data were collected through a data collection, and audio recordings were made to complete and validate the data collection form afterwards. The data collection form included topics such as contact with the informal caregiver, privacy, WWHAM questions, information provision, recommendations, the reason for referral, dispensing and conclusion. The form included closed questions with dichotomous answer options, closed multiple-choice, and open questions. AMP stored the audio files.

#### 2.2.2 Data analysis

The data collected were analyzed using IBM SPSS statistics for Windows version 29.0.2.0. Different consultation elements were analyzed and grouped including general consultation characteristics, WWHAM questions, and recommendations, which were split into general recommendations, GP referral, sick day guidance and product dispensing. Providing adequate recommendations was defined as either providing sick day guidance recommendations during the consultation or if a referral to the GP was recommended. A Mann-Whitney *U* test was performed to asses significance between adequate and non-adequate consultation duration. Lastly, the relationship between asking about comorbidities and current medications use and consultation recommendations was assessed. The data were analyzed descriptively and depicted as a median with an interquartile range (IQR) or counts including a percentage. The research is reported according to CRiSPHe, a checklist for reporting research using a simulated patient methodology in Health, see Appendix B.^8^

## 3 Results

A total of 64 pharmacies participated, of which 62 were community pharmacies and two were outpatient pharmacies in a hospital. The participating pharmacies were spread across the Netherlands and varied in size and setting, ranging from small towns to bigger cities, see Ap.

The median waiting time before the consultation was two (IQR: 1–6) minutes, and the median consultation duration was three (IQR: 2–6) minutes. In five pharmacies, a colleague was consulted to help with the self-care inquiry, of whom one was a pharmacist. More than 70 % of the pharmacies identified the person experiencing diarrhoea and asked how long the complaints had been present. Half of the pharmacies mentioned the risk of dehydration due to diarrhoea.

In 25 % of the pharmacies, adequate recommendations were provided, either by recommending temporary medication adjustments directly during consultation (two pharmacies), through referral to the GP for further advice (eight pharmacies), or by providing both (six pharmacies). (The median consultation time for the pharmacies providing inadequate recommendations was three minutes, compared to five and a half minutes for the pharmacies providing adequate recommendations, the difference was statistically different (*p* *=* *0,01).*

A product was dispensed in 53 out of the 64 pharmacies, most often loperamide, followed by oral rehydration salts. Activated charcoal was dispensed once, see Table 1.

**Table 1:** Summary of consultation performance.

	Total number of pharmacies (*n* = 64)
General consultation characteristics	
Waiting time before consultation, median in minutes (IQR)	2 (1–6)
Consultation time, median in minutes (IQR)	3 (2–6)
Sufficient privacy during consultation, n (%)	36 (56.3)
Asked for identification to register in the pharmacy information system, n (%)	36 (56.3)

WWHAM questions	
Identification of who is experiencing diarrhoea, n (%)	45 (70.3)
Asked how long the complaints are present, n (%)	47 (73.4)
Asked whether other things have already been tried to solve the complaints, n (%)	39 (60.9)
Asked about comorbidities and other medications, n (%)	38 (59.4)
Specific question on laxative use, n (%)	1 (1.6)

Recommendations	
General recommendations	
Mentioned risk of dehydration caused by diarrhoea, n (%)	33 (51.6)
Fluid intake recommendations, n (%)	21 (32.8)
Food intake recommendations, n (%)	13 (20.3)
Risk of NSAID use, n (%)	1 (1.6)
Provided additional information materials, n (%)	14 (21.9)

Referral to general practitioner	
Patient referred to GP, n (%)	14 (21.9)
Due to age, n (%)	7 (10.9)
Due to diabetes comorbidity, n (%)	5 (7.8)
Due to other medications in use, n (%)	11 (17.2)
Due to the need for additional advice on medications and dehydration, n (%)	8 (12.5)
Always refer to GP in case of diarrhoea, n (%)	1 (1.6)

Sick day guidance	
Sick day guidance was applied, n (%)	8 (12.5)
Temporarily discontinue metformin, n (%)	5 (7.8)
Temporarily discontinue perindopril, n (%)	2 (3.1)
Discuss temporary adjustment with GP, n (%)	5 (7.8)

Product dispensing	
Product dispensed	53 (82.8)
Oral rehydration salts, n (%)	10 (15.6)
Loperamide, n (%)	42 (65.6)
Activated charcoal, n (%)	1 (1.6)

IQR: inter quartile range, WWHAM: Who is it for, What are the symptoms, How long have the symptoms been present, has any Action been taken, what Medications are in use, NSAID: non-steroidal anti-inflammatory drug, GP: general practitioner.

**Fig. 2:**
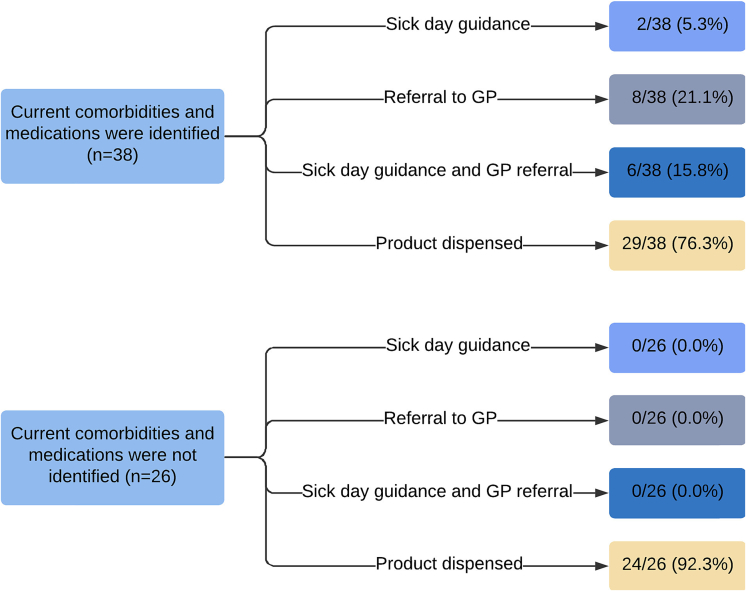
Relationship between the consultation recommendations and whether pharmacies asked about current comorbidities and medications in use during consultation.

Thirty-eight of 64 pharmacies asked about current comorbidities and other medications of the elderly relative. None of the pharmacies that did not ask about comorbidities and other medications, provided sick day guidance or referred to the general practitioner. Additionally, pharmacies that did not ask about other comorbidities and medications more often dispensed a self-care product (92 %) compared to pharmacies that did ask about current comorbidities and medications (76 %), see Fig. 2.

## 4 Discussion

This simulated patient study showed that although current comorbidities and medications were identified in more than half of the pharmacies, only one out of eight community pharmacies applied sick day guidance when a simulated informal caregiver of a relative with a potential risk of acute kidney failure visited the pharmacy for advice to treat diarrhoea. In 22 % of the cases, a referral to the GP was made. No referrals or sick day guidance recommendations were applied in the pharmacies that did not ask about current comorbidities and medications.

The results show that only 25 % of the pharmacies provided sick day recommendations according to the clinical guideline for self-care. Providing sick day recommendations or referring patients to a GP depended on whether the current comorbidities and medications were identified. Without this information no appropriate sick day guidance or referral was provided, leading to underestimation of the situations' severity, potentially harming patients. Even when the current comorbidities and medications were identified, over half of the pharmacies still failed to provide guidance or referrals, indicating pharmacy staff's unfamiliarity with sick day guidance and supporting previous findings of limited awareness on the prevention of acute kidney injury among healthcare providers.^9^^,^^10^ Therefore, when the pharmacy refers a patient to the GP, they should also mention explicitly why they refer them so the patient can communicate this to the GP assistant. Otherwise, it will be likely that the GP assistant is unaware of sick day guidance and as a consequence, does not provide adequate recommendations as well.

Despite Dutch guidelines indicating that no drug therapy is indicated in most cases of acute diarrhoea, four out of five pharmacies in this study dispensed a self-care product. This may stem from the caregiver's initial request for a diarrhoea remedy, making it difficult for staff to send them away empty-handed. Additionally, while the guideline states that elderly patients have a higher dehydration risk, no specific medication advice is provided for this group. It is reasonable for the participating pharmacies to dispense a product for this high-risk group. Products like oral rehydration salts or loperamide are generally safe when used correctly. It is recommended that future guidelines indicate that patients in whom dehydration poses an increased risk should not only be referred to the GP but should first and foremost be advised to promote rehydration by using ORS.

Only one pharmacy specifically asked whether or not the elderly relative was using laxatives, and only one pharmacy explicitly warned against NSAID use. This is partly due to the caregiver not considering macrogol (laxative) a medication and the absence of pain or fever that could be a reason for NSAID use. However, it may be prudent to routinely inquire about laxative use during diarrhoea and NSAID use in high-risk patients to prevent kidney injury. Additionally, this study showed that investing only two and a half minutes extra through additional questions in the consultation already results in more adequate recommendations for the patient. However, this finding should be interpreted with caution, because in clinical practice, patient complexity can differ from the case used in this study. Additionally, staff shortages and a high workload in the participating pharmacies could have influenced the consultation time recorded.

A strength of this study was that the simulated informal caregivers were well-trained and the case was thoroughly studied before being put into practice. This reduces bias and provides an objective measure of quality of care. A limitation of the study is that it is unknown whether the participating pharmacist had communicated the topic of the case to the pharmacy team beforehand. If so, the pharmacy employees could have prepared themselves and therefore, the results might have been inflated. On the other hand, sick day guidance was not explicitly mentioned in the announcement and many pharmacies currently are understaffed and face time-consuming issues with drug shortages, which makes it less likely that they prepared intensively for this visit. Additionally, the pharmacies participating in the AMP simulated patient programme are potentially more ambitious and acknowledge the value of good self-care management. Therefore, the quality of care could be overestimated compared to the average pharmacy in the Netherlands. Lastly, the generalizability of these findings may be limited to pharmacies in the Netherlands as guidelines and practice models can differ between countries.

In conclusion, although half of the pharmacies mentioned dehydration risks, only one out of four pharmacies provided sick day guidance or referred to a general practitioner. Asking about current comorbidities and medications in use during consultation is crucial in providing adequate recommendations to patients with self-care questions.

## CRediT authorship contribution statement

**Tristan Coppes:** Writing – review & editing, Writing – original draft, Visualization, Validation, Supervision, Software, Resources, Project administration, Methodology, Investigation, Formal analysis, Conceptualization. **Hetty Prins:** Writing – review & editing, Writing – original draft, Resources, Methodology, Investigation, Data curation. **Kees A. van Amerongen:** Writing – review & editing, Writing – original draft, Visualization, Validation, Resources, Investigation, Data curation, Conceptualization. **Teun van Gelder:** Writing – review & editing, Writing – original draft, Visualization, Supervision, Conceptualization. **Ellen S. Koster:** Writing – review & editing, Writing – original draft, Visualization, Supervision. **Marcel L. Bouvy:** Writing – review & editing, Writing – original draft, Visualization, Validation, Supervision, Conceptualization.

## Ethical approval

All data were anonymised and could not be traced back to the participating pharmacies. All pharmacists participating in the assessment program have provided written consent to AMP to use the assessment data for research.

## Funding

No funding was received for this study.

## Declaration of competing interest

KvA reports a relationship with Stichting Uitgifte Informatie that includes: board membership. In the last 3 years, TvG has received lecture fees and consulting fees from Roche Diagnostics, Thermo Fisher, Vitaeris, Otsuka, CSL Vifor, Astellas and Aurinia Pharma. In all cases money has been transferred to hospital accounts, and none has been paid to his personal bank accounts. The authors declare that they have no known competing financial interests or personal relationships that could have appeared to influence the work reported in this paper.

## Data Availability

The data and the data collection form are available upon request and subject to AMP approval.

## Appendix A Supplementary data

Appendix A: Detailed case description. Image 1

Appendix B: Checklist for reporting research using a simulated patient methodology in Health (CRISPHE). Image 2

Appendix C: Overview table of participating pharmacies. Image 3
