# Supplementary material for: Using simulated patient methodology to assess sick day guidance in community pharmacy: The case of an elderly patient with diabetes
Source: Explor Res Clin Soc Pharm. 2025 Jun 11;19:100623. doi: 10.1016/j.rcsop.2025.100623 (PMC12210287; doi:10.1016/j.rcsop.2025.100623)
Supplement: Supplementary file 1 — Appendix A: Detailed case description. [file mmc1.pdf]

Appendix A: Detailed case description

Case description

An informal caregiver visits the pharmacy to ask for advice about diarrhoea for a 71-year-old family member with type 2 diabetes. The patient lives on his own. He is being monitored by his GP's practice assistant for diabetes. The caregiver knows that his kidney function is periodically monitored, but does not know the value. The patient has had watery diarrhoea 6 times a day for one day. He is not otherwise ill, has no fever, is not nauseous and is not vomiting. The patient doesn't think it necessary to do anything about it. He thinks it will pass. Nevertheless this simulated caregiver is worried and now asks for advice at the pharmacy.

Other comorbidities of the patient include hypercholesterolemia and hypertension for which he uses medication. Lastly, the patient also uses macrogol sachets daily to loosen the stool. But the caregiver does not consider macrogol as medication so this is only mentioned if explicitly asked by the pharmacy.

The first interaction between the informal caregiver in the pharmacy is: “I would like to get something for diarrhoea…”. No other information is spontaneously provided to the pharmacy by the simulated informal caregiver.

Medication	Prescription
Metformin	500 mg 2 tablets twice a day (during breakfast and dinner)
Simvastatin	20 mg once per day (evening)
Perindopril	4 mg once per day (with breakfast)
Amlodipin	5 mg once per day (evening)
Macrogol sachets	1x per day (morning) (was only mentioned if explicitly asked)

Spontaneous Information

“I would like something for diarrhoea…”

Guided Information

(*Only provided by simulated informal caregiver when asked)*

- Is it for yourself?
No, it’s for my brother/brother-in-law/...
- Who is it for/who is suffering?
My brother/brother-in-law...
- What is his age?
71 years
- What is his date of birth?
Between July 1951 and April 1952 (e.g., February 14, 1952).
- What is the issue?
Oh, it’s not for me, it’s for my brother/brother-in-law...
- What is he experiencing?
Diarrhoea
- How long has this been an issue?
Since yesterday/1 day

- Has it lasted more than 3 days?
No, only since yesterday/1 day
- Did it start suddenly?
Yes
- **Has he had diarrhoea before?**
No
- What does the stool look like?
Watery
- Is it completely watery?
Yes, definitely
- Does it contain blood or mucus?
No
- How frequent is the stool?
Every few hours
- Is it more than 6 times a day?
Yes, definitely, maybe even more...
- Is he nauseous or vomiting?
No
- Does he have abdominal cramps?
No
- Does he have flatulence?
No
- Does he have abdominal pain?
No
- Does he know what caused it?
No
- Could it be from something he ate or drank?
Possibly, but I don't know
- Has he travelled abroad/to tropical regions recently?
No
- Are other family members/housemates/neighbours also experiencing diarrhoea?
No
- Has he taken anything for the diarrhoea?
No
- Is he drinking enough fluids?
I don't know
-Does he have other conditions?
yes he has diabetes

Other Conditions

- Does he have diabetes?
Yes
- Does he have kidney problems or impaired kidney function?
I don't know
- Is the kidney function known?
It's monitored, but I don't know the value
- Does he have other conditions?
High cholesterol and high blood pressure
- Does he have...
- Digestive disease (e.g., Crohn's, IBS, lactose intolerance)? No
- Heart failure? No
- Weak immune system? No

Medication

- Does he use medication?
Yes, for diabetes, high cholesterol, and high blood pressure.
- What medication?
(See overview above)
- Does he use insulin?
No
- Does he take tablets for diabetes?
Yes, metformin, prescribed by the general practitioner
(SGLT2 inhibitors, No)
- Does he use a laxative?
Yes, macrogol sachets
- if asked, the patient does NOT use any of the following:
Antibiotics
Magnesium
Methotrexate
Colchicin
Theofyllin
Anti-epileptics
Lithium
Digoxin
- Has there been any recent change in medication?
No
-How long has he been using metformin?
Almost 3 years
-Has he been hospitalized recently?
no
- Do they monitor blood glucose levels?
Yes

General Condition

- Does he show dehydration symptoms?
I'm not sure
- Does he look ill?
I can't assess that well
- Is he alert?
Yes
- Is he pale/clammy, have a rapid pulse, or have no appetite?
No
- Has he consulted a doctor?
No
-Does he use self-care medication?
No, except a painkiller sometimes
-What kind of painkiller?
I am not sure, I think paracetamol
-Does he use NSAIDs?
I don't know
