# Supplementary material for: Using simulated patient methodology to assess sick day guidance in community pharmacy: The case of an elderly patient with diabetes
Source: Explor Res Clin Soc Pharm. 2025 Jun 11;19:100623. doi: 10.1016/j.rcsop.2025.100623 (PMC12210287; doi:10.1016/j.rcsop.2025.100623)
Supplement: Supplementary file 2 — Appendix B: Checklist for reporting research using a simulated patient methodology in Health (CRISPHE). [file mmc2.pdf]

Appendix B: CRISPHE checklist

**CRiSPHe: Checklist for reporting research using a simulated patient methodology in Health**		
Title and Background	1 Include the term simulated patient (e. g. mystery shopper) in the title, abstract or key words. **Use the MeSH heading 'Patient Simulation'.**	Simulated patient was added.
	2 Describe the rationale, theory, or goal behind using simulated patient methodology.	The goal was to objectively assess clinical guideline adherence for a self-care question in the community pharmacies.
	3 Report the study design used (e.g. cross-sectional, case-control, randomized controlled trial).[*]	Cross-sectional
Simulated Patients	4a Report the number of simulated patients used in the study.	3
	4b If more than one simulated patient was used, describe methods used to minimize variability between simulated patients.	An elaborate case file was developed with standardized information and answer sheets for possible questions.
	5 Report demographics of the person playing the simulated patient(s) **role** such as age, **sex and/or gender identity**, qualifications (e.g. student, academic), characteristics relevant to the scenario (e.g. has acne for an acne scenario), **and simulated agents of patients (e.g. tretinoin for acne).**	The person who visited the pharmacy acted as an informal caregiver (brother/sister (in-law). The person is a trained actor with experience in simulated patient studies in pharmacies.
	6 Describe what was done during training sessions for simulated patients (e.g. role-play scenarios).	
	7 If simulated patient fidelity was assessed, describe the extent to which the scenario was delivered as planned. **Describe how the fidelity was determined.**	Fidelity was not assessed.
Simulated Patient Scenarios	8a Describe how the scenarios were developed (including who they were developed by).	The scenario was developed by AMP, an organization experienced in performing simulated patient studies. During the development, experts on the topic were

		consulted to reflect on the scenario.
	8b Describe which guidelines (i.e. **standard** best practice guidelines) used in development of the scenarios **if any** or, if not, describe how the scenarios were validated.	The Royal Dutch Pharmacy Association (KNMP) self-care guideline for acute diarrhoea was used for development.
	9a Outline the scenario(s) used. Include any patient characteristics, patient prompts, scripts, props (e. g. prescriptions, medical devices).	See Appendix 1.
	9b Describe any flexibility in scenarios or scripts to allow simulated patients to adapt based on participant responses.	See Appendix 1.
	10a Materials: Describe any physical or informational materials used in the simulated patient encounter, including those provided to participants or used during the SP encounter or in training of SPs. Provide information on where the materials can be accessed (e.g. online appendix[/scolor], URL).	See Appendix 1.
	10b Include a copy of any scripts or material given to simulated patients. E.g. include a full copy of any script for SP scenario, copies of actual prescri<br><br>ptions, pamphlets, props (e.g. inhaler device) used by SPs during the SP encounter.	See Appendix 1.
	11 Describe any intervention/ activity completed prior to the simulated patient encounter. Include procedures, activities, and/ or processes (e.g. training sessions for health professionals).	The pharmacists received information regarding the topic of the simulated patient scenario a few weeks beforehand. The pharmacists could decide whether they would reveal the topic to the pharmacy team before the visitation.
	12 Describe each of the procedures, activities, **environmental context,** and/ or processes **in full** used for the simulated patient encounters, including any enabling or support activities.	No additional procedures were present.
	13 If the simulated patient assessment was modified (e.g., changes in personnel, assessment rubric, patient history or	No changes added.

	problems), describe these changes (what, why, when, and how).	
	14 Describe any procedures that followed the simulated patient assessment (e.g. debriefs, performance feedback), including how (face to face, phone) and when these were conducted.	An evaluation report is made afterwards. All participating pharmacies' data forms are collected and the average, best and worst performances are reported back anonymously to individual pharmacies as a benchmark. The report also included their performances.
	15 Describe plans for incidents where the simulated patients were identified by participants as a simulated patient. Report if identification of the SP was made.	If the simulated patient suspected that the pharmacy was aware of the simulation, this was notified to the researchers. Data would still be included in this study. No reports were made.
Data Collection	16 Report how many simulated patient visits were conducted (include the planned number of visits, the number of actual completed visits, the number of visits per SP, number per scenario, number per health services provider e.g. pharmacy).	Total number of visits: 64<br>Total number of participating pharmacies: 64<br>Number of (average) visits per simulated patient: 21
	17 Describe the mode(s) of delivery of the simulated patient assessment (e.g., face-to-face, telephone, internet, text, live, asynchronous).	Face-to-face in the pharmacy
	18 Describe the data collection procedure (e.g. data collection form, audio recording, telephone calls).	Data collection form and audio recording for validation.
	19 Describe how any data collection forms were created and validated (include a copy of any data collection forms if possible).	The data collection form is created by AMP and is tested once in pilot testing. The form is validated afterwards by AMP with the audio recording.
	20 Describe when the data was collected by the simulated patient (i.e. during the visit, immediately after).	During the visit and completed immediately after.

		Validated with audio afterwards.
	21 Report ways to avoid or minimize recall bias (e.g. if immediately recorded after encounter, use of audiotaping, use of a **second** observer, **use of a checklist)**	Recall bias was minimized by directly filling in the form and validating the form with an audio-recording.
	22 Report any potential conflicts of interest (e.g. if a simulated patient is a student assessing a colleague or preceptor; funding source [if a simulated patient is a student])	No conflict of interests.
Ethics	23a Describe any **ethical and/or organizational** approvals gained prior to conducting the research.	Through subscription with the AMP simulated patient scenarios, the pharmacies approved to be visited 4 times a year and using the results in scientific research.
	23b Describe the processes for gaining consent from participants and ways of maintaining confidentiality.	Through subscription with the AMP simulated patient scenarios, the pharmacies approved using the results in scientific research. If the data is shared with an external party such as a University. A data-sharing form is signed to secure confidentiality and anonymity.
	23c Explain how participants were informed about being assessed using covert methods. If they were not, justify this.	The participating pharmacies are subscribed to the AMP simulation service voluntarily and are aware of the used methods.
