# Supplementary material for: Using simulated patient methodology to assess sick day guidance in community pharmacy: The case of an elderly patient with diabetes
Source: Explor Res Clin Soc Pharm. 2025 Jun 11;19:100623. doi: 10.1016/j.rcsop.2025.100623 (PMC12210287; doi:10.1016/j.rcsop.2025.100623)
Supplement: Supplementary file 3 — Appendix C: Overview table of participating pharmacies. [file mmc3.pdf]

Appendix C: Overview table of participating pharmacies

Table 1: Overview of the number of inhabitants of the towns or cities where the pharmacies were situated:

	Total number of pharmacies (n=64)
Pharmacy located in, n(%)	
Small town: < 20.000 inhabitants	15 (23)
Medium-sized town: 20.000 - 49.999 inhabitants	12 (19)
Large town: 50.000 - 99.999 inhabitants	14 (22)
Small city: 100.000 - 199.999 inhabitants	15 (23)
Medium city: 200.000 - 499.999 inhabitants	1 (2)
Big city: > 500.000 inhabitants	7 (11)
